# Management of Hypertension in Chronic Kidney Disease

**DOI:** 10.1007/s40265-019-1064-1

**Published:** 2019-02-13

**Authors:** Dan Pugh, Peter J. Gallacher, Neeraj Dhaun

**Affiliations:** 1 University/BHF Centre for Cardiovascular Science, The Queen’s Medical Research Institute, University of Edinburgh, 47 Little France Crescent, Edinburgh, EH16 4TJ Scotland UK; 2 Department of Renal Medicine, Royal Infirmary of Edinburgh, Edinburgh, UK

## Abstract

Chronic kidney disease (CKD) is an increasingly prevalent condition globally and is strongly associated with incident cardiovascular disease (CVD). Hypertension is both a cause and effect of CKD and affects the vast majority of CKD patients. Control of hypertension is important in those with CKD as it leads to slowing of disease progression as well as reduced CVD risk. Existing guidelines do not offer a consensus on optimal blood pressure (BP) targets. Therefore, an understanding of the evidence used to create these guidelines is vital when considering how best to manage individual patients. Non-pharmacological interventions are useful in reducing BP in CKD but are rarely sufficient to control BP adequately. Patients with CKD and hypertension will often require a combination of antihypertensive medications to achieve target BP. Certain pharmacological therapies provide additional BP-independent renoprotective and/or cardioprotective action and this must be considered when instituting therapy. Managing hypertension in the context of haemodialysis and following kidney transplantation presents further challenges. Novel therapies may enhance treatment in the near future. Importantly, a personalised and evidence-based management plan remains key to achieving BP targets, reducing CVD risk and slowing progression of CKD.

## Key Points

**Table:** 

Controlling hypertension in those with chronic kidney disease (CKD) not only slows progression of renal damage but reduces the risk of cardiovascular disease.
Achieving blood pressure (BP) control in CKD may be difficult, often requiring a combination of antihypertensive medications as well as lifestyle modifications.
One size does not fit all—an understanding of the existing evidence is vital in order to deliver personalised management and achieve BP targets.

## Introduction

Chronic kidney disease (CKD) affects 10–15% of the population worldwide and its prevalence is increasing [1, 2]. CKD is defined as the presence of reduced kidney function (an estimated glomerular filtration rate [eGFR] < 60 mL/min/1.73 m^2^ [3]) or kidney damage (often indicated by the presence of proteinuria) for ≥ 3 months duration [4]. Hypertension, defined by the European Society of Cardiology and the European Society of Hypertension (ESC/ESH) as a blood pressure (BP) of ≥ 140/80 mmHg affects ~ 30% of the general adult population and up to 90% of those with CKD [5, 6].

Hypertension is both a cause and effect of CKD and contributes to its progression [7–9]. As eGFR declines, the incidence and severity of hypertension increase [5]. Additionally, hypertension and CKD are both independent risk factors for cardiovascular disease (CVD). When both exist together the risks of CVD morbidity and mortality are substantially increased [10]. For those with stage 3 (eGFR 30–59 mL/min/1.73 m^2^) or stage 4 (eGFR 15–29 mL/min/1.73 m^2^) CKD, defined according to the Kidney Disease: Improving Global Outcomes (KDIGO) guidelines [4], the risk of death due to CVD is higher than the risk of progression to end-stage renal disease (ESRD) (eGFR < 15 mL/min/1.73 m^2^) [11, 12]. Importantly, from a therapeutic perspective, lowering BP can slow eGFR decline, delay progression to ESRD, and reduce the incidence of CVD in this patient group [13, 14].

## Pathogenesis of Hypertension in Chronic Kidney Disease (CKD)

**Fig. 1:**
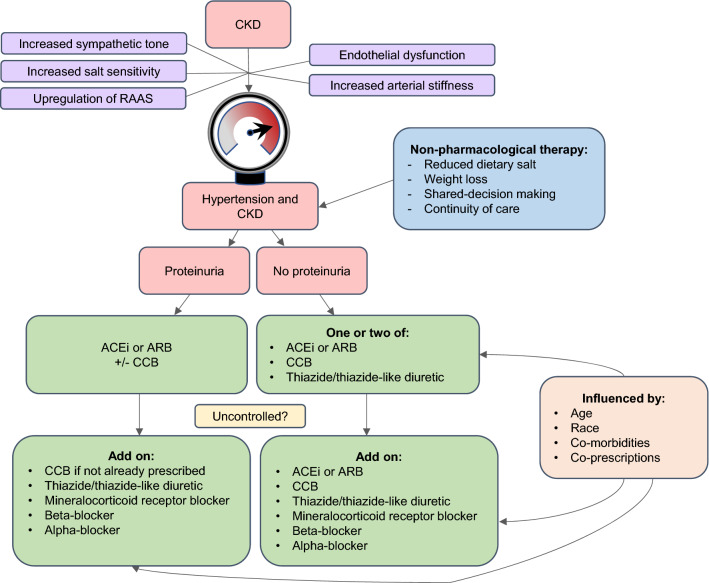
Pathogenesis and management flow-chart of hypertension in chronic kidney disease. *ACEi* angiotensin converting enzyme inhibitor, *ARB* angiotensin II receptor antagonist (blocker), *CCB* calcium channel antagonist (blocker), *CKD* chronic kidney disease, *RAAS* renin–angiotensin–aldosterone system

A number of mechanisms contribute to the development of hypertension in CKD and these influence its management (Fig. 1). Increase in sympathetic tone, brought about by afferent signals generated by functionally declining kidneys, contributes to the development of hypertension in CKD [15]. As eGFR declines there is an upregulation of the renin–angiotensin–aldosterone system (RAAS) which promotes salt and water retention [16]. This is compounded by an increased salt sensitivity of BP [17]. Endothelial dysfunction is characteristic of advanced CKD (eGFR < 30 mL/min/1.73 m^2^) and its association with hypertension is well-established [18]. Increased arterial stiffness is also seen throughout the spectrum of CKD [19], is implicated in the development of hypertension [20], and is an independent risk factor for CVD events [21]. Once hypertension has developed, several factors, including increased oxidative metabolism, with resultant relative renal hypoxia, may drive further progression of BP and CKD [22, 23].

In health, BP demonstrates a nocturnal dip of ~ 10 to 20%. This is controlled by several factors including diurnal variations in autonomic function, salt excretion and the RAAS [24]. Dysregulation of these systems in CKD leads to a non-dipping or even rising nocturnal BP, which is associated with increased CVD morbidity and mortality and risk of CKD progression [25, 26].

## Measurement of Blood Pressure (BP) in CKD

For management of hypertension to be effective, accurate BP measurements are essential. In practice, the treatment of hypertension is often based on clinic or office BP recordings [27]. These may be inaccurate due to lack of repeat measurements, diurnal variation in BP and white-coat hypertension [28]. Thus, this snapshot of BP may not accurately define the clinical problem. Different phenotypes of hypertension are recognised and associated with varying degrees of CVD risk (Table 1). In order to identify these and institute treatment accordingly, more robust methods of measuring BP should be used.

**Table 1:** Association of hypertension phenotype with all-cause mortality (adapted from Banegas et al. [33] using Cox regression model)

BP Phenotype	Description^a^	All-cause mortality hazard ratio (95% CI)
Normotension	Normal clinic BP, normal 24-h ABPM	Reference
White-coat hypertension	High clinic BP, normal 24-h ABPM	1.79 (1.38–2.32)
Sustained hypertension	High clinic BP, high 24-h ABPM	1.80 (1.41–2.31)
Masked hypertension	Normal clinic BP, high 24-h ABPM	2.83 (2.12–3.79)

Values represent patients on treatment and without chronic kidney disease

*ABPM* ambulatory blood pressure monitoring, *BP* blood pressure, *CI* confidence interval

^a^Normal clinic BP defined as < 140/90 mmHg. Normal 24-h BP defined as < 130/80 mmHg

24-Hour ambulatory BP monitoring (ABPM) provides a more accurate depiction of BP phenotype and is a better predictor of CVD events in those with CKD than clinic readings [29]. 24-Hour ABPM also allows assessment of the diurnal variation in BP. Home BP monitoring is an alternative strategy that is less resource intensive. Those who obtain home readings demonstrate better overall BP control than those who do not [30]. Current hypertension guidelines reflect this, with the 2017 American College of Cardiology (ACC) guidelines supporting out-of-office BP measurement to confirm the diagnosis of hypertension and for titration of BP-lowering medication in all patients [31]. To ensure accuracy, only validated home BP devices should be used [32]. ACC guidelines also describe the anticipated relationship between clinic and out-of-clinic BP measurements, suggesting that a clinic BP of 140/90 mmHg equates approximately to a home BP value of 135/85 mmHg and to daytime and nocturnal ABPM values of 135/85 and 120/70 mmHg, respectively [31].

## Proteinuria

Proteinuria is an important marker of renal damage and is incrementally and independently associated with CKD progression and incident CVD [10]. Quantification of proteinuria allows stratification of this risk and can also be used as a marker of response to treatment (Table 2). The most practical way to measure proteinuria is with a protein-to-creatinine ratio (PCR) obtained from a spot urine sample. An albumin-to-creatinine ratio (ACR) is more accurate when protein leak is minimal, with an ACR value of ≥ 3 mg/mmol sufficient for a diagnosis of CKD regardless of eGFR [34]. Total daily proteinuria can be obtained via a 24-h urine collection or extrapolated from a PCR or ACR measurement [35]. Although 24-h urine collection remains the gold standard method for quantification of proteinuria, susceptibility to patient and sampling errors can lead to inaccuracies [36]. Several studies have demonstrated equivalency or superiority of ACR or PCR over 24-h albumin or protein excretion in predicting CKD progression [37, 38].

**Table 2:** Quantification of proteinuria (adapted from Kidney Disease: Improving Global Outcomes 2012 chronic kidney disease guidelines [4])

Quantification method	Normal or mildly increased	Moderately increased	Severely increased	Nephrotic range
Dipstick	Negative to trace	Trace to +	+ or greater	+++ or greater
ACR				
mg/mmol	< 3	3–30	> 30	> 220
mg/g	< 30	30–300	> 300	> 2200
PCR				
mg/mmol	< 15	15–50	> 50	> 300
mg/g	< 150	150–500	> 500	> 3000
24-h urinary protein (g/day)	< 0.15	0.15–0.5	> 0.5	> 3

Relationship between measurement methods are not exact and will depend on multiple variables

*ACR* albumin-to-creatinine ratio, *PCR* protein-to-creatinine ratio

BP reduction reduces proteinuria, which slows eGFR decline and reduces CVD [39]. More intense BP reduction (a target systolic BP < 120 mmHg) may offer greater renoprotection in those with significant proteinuria (> 1 g/day; PCR > 100 mg/mmol, ACR > 70 mg/mmol) than in those without proteinuria [40, 41]. In addition to its antihypertensive effects, the impact of a drug on proteinuria is an important consideration when managing hypertension in CKD. In particular, RAAS blockade appears to offer a BP-independent reduction in proteinuria [42]. Thus, these medications are considered first-line therapy for those with proteinuric CKD [31].

## Goals of BP Reduction and BP Targets

Guidelines offer the treating clinician a rapid, evidence-based, expert opinion regarding the management of certain conditions. Often criticised for a lack of flexibility, however, they are seen by some as unhelpful due to the complexities involved in clinical decision-making. Guidelines governing the management of patients with CKD are relatively few in relation to other conditions of similar prevalence. This may, in part, reflect the relative dearth of high-quality clinical trials in CKD. Despite this, guidelines outlining optimal treatment for CKD patients with hypertension are important, particularly as many of these patients are jointly managed in primary care.

**Fig. 2:**
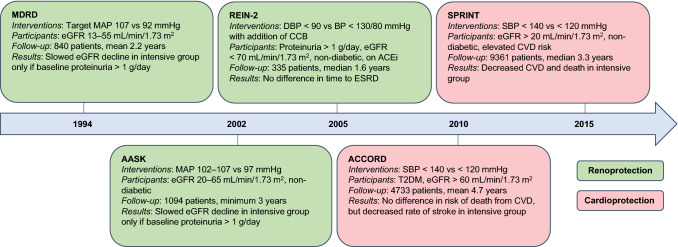
Timeline of landmark randomised trials comparing standard with intensive blood pressure control. *ACEi* angiotensin converting enzyme inhibitor, *BP* blood pressure, *CCB* calcium channel antagonist (blocker), *CVD* cardiovascular disease, *DBP* diastolic blood pressure, *eGFR* estimated glomerular filtration rate, *ESRD* end-stage renal disease, *MAP* mean arterial pressure, *MDRD* Modification of Diet in Renal Disease, *SBP* systolic blood pressure, *T2DM* type 2 diabetes mellitus

In their 2017 guidelines, the ACC recommended that all adults with hypertension and CKD should be treated to a target BP of < 130/80 mmHg regardless of proteinuria [31]. The National Institute for Health and Care Excellence (NICE) and UK Renal Association suggest a more conservative target of < 140/90 mmHg, provided proteinuria is < 1 g/day [43, 44]. In the presence of greater degrees of urinary protein leak this target is revised to < 130/80 mmHg. KDIGO guidance also suggests a lower BP target for those with significant proteinuria, although it deploys a cut-off of > 300 mg/day [45]. The 2018 ESC/ESH guidelines suggest a target systolic BP of < 140 mmHg regardless of proteinuria [46]. To understand the differences between these guidelines one must consider the evidence used to create them (Fig. 2).

## BP Control for Renal Protection

The Modification of Diet in Renal Disease (MDRD) study was the first randomised trial to investigate the effect of standard (target mean arterial pressure [MAP] 107 mmHg) versus intensive (target MAP 92 mmHg) BP control on the rate of eGFR decline in a US population with CKD (eGFR 13–55 mL/min/1.73 m^2^). In patients with baseline proteinuria > 1 g/day intensive BP control did slow the rate of eGFR decline when compared with standard BP control. However, no such benefit was seen in those without proteinuria [40]. In 2002, AASK (African American Study of Kidney Disease and Hypertension) mirrored these results in a non-diabetic African American population with a GFR of 20–65 mL/min/1.73 m^2^. In this study, 1094 patients were randomised to receive either standard (MAP 102–107 mmHg) or intensive (MAP 97 mmHg) BP control with a minimum follow-up period of 3 years. Again, only patients with baseline proteinuria > 1 g/day demonstrated slowing of CKD with intensive BP control [41, 47]. The REIN-2 (Ramipril Efficacy in Nephropathy-2) study examined whether intensive BP control with the addition of a dihydropyridine calcium channel antagonist (blocker) (CCB) to those already established on an angiotensin converting enzyme (ACE) inhibitor was superior to standard BP control with an ACE inhibitor alone. The study included those with CKD and baseline proteinuria > 1 g/day. The addition of a CCB did reduce BP; however, this did not translate into improved renoprotection [48]. Guidelines published in the wake of these landmark studies reflected these results, suggesting lower targets only for those with significant proteinuria. However, these studies did not consider the potential benefits of intensive BP control on cardiovascular endpoints.

## BP Control for Cardiovascular Protection

The ACCORD (Action to Control Cardiovascular Risk in Diabetes) study examined the effect of intensive (target systolic BP < 120 mmHg) versus standard (target systolic BP < 140 mmHg) BP control on cardiovascular outcomes (myocardial infarction [MI], stroke or death from CVD) in patients with type 2 diabetes mellitus and normal renal function (serum creatinine < 133 μmol/L) [49]. Despite a sustained difference in attained BP between the standard and intensive treatment groups, the risk of death from CVD did not differ significantly. There was, however, a reduced risk of stroke with intensive BP control.

Published in 2015, SPRINT (Systolic Blood Pressure Intervention Trial) compared a systolic BP target of < 140 mmHg with a more intensive systolic goal of < 120 mmHg in 9361 non-diabetic patients at elevated CVD risk (defined as the presence of CVD, CKD, age > 75 years, or 10-year CVD risk ≥ 15%) [50]. The intensive treatment group demonstrated a statistically significant reduction in the primary outcome, a composite of MI, acute coronary syndrome, stroke, heart failure or death from CVD. A reduction in the secondary outcome of death from any cause was also significant. The effect size was so large the trial was stopped prematurely after three of its projected 5-year duration. In those with CKD (*n *= 2646, mean GFR 47.9 mL/min/1.73 m^2^) intensive BP control reduced all-cause mortality with an effect size similar to that seen in the overall cohort [14]. Despite this, there was no effect on renal outcomes, including the rate of eGFR decline. Unfortunately, those with eGFR < 20 mL/min/1.73 m^2^ and/or proteinuria > 1 g/day were excluded from SPRINT, as were those with diabetes, who account for up to 45% of CKD in the developed world [51]. Nevertheless, SPRINT suggests that intensive BP control reduces CVD morbidity and mortality in those with CKD. Of note, extended follow-up data now available from both the MDRD and AASK studies also suggest a long-term survival benefit from intensive BP lowering despite no change in the rate of CKD progression [52, 53].

It should be noted that the method of BP measurement used may have a significant impact on the outcome of any trial. In SPRINT, BP was measured after participants were seated in a quiet room for 5 min with no distractions. SPRINT BPs may, therefore, not be directly comparable to our clinic BPs, which are likely to be higher. This may be why such a low target BP was found to be beneficial. Notably, the risk of developing acute kidney injury (AKI) was increased in the intensive treatment group. Additionally, in those with normal renal function at baseline, the risk of developing CKD during the study was higher with intensive treatment. An analysis by Zhang et al. [54] has investigated whether these changes represent true renal injury by examining biomarkers of kidney damage in this cohort. They found that the intensive BP lowering used in SPRINT was associated with a *decrease* in kidney damage biomarkers, including ACR, anti-chitinase-3-like protein 1 and uromodulin, suggesting that benign alteration in renal blood flow, rather than pathological processes, may be responsible for the changes in eGFR observed [54].

The available evidence does not provide a clear consensus regarding the optimal BP target in CKD. Accordingly, guidelines vary. The 2017 ACC guidelines employed the results of SPRINT as the basis for a more intensive BP target. However, the 2018 ESC/ESH guidelines have not adopted this more intensive approach. Perhaps this could be interpreted as a prioritisation of cardiovascular protection over renoprotection by the Americans, and vice versa by the Europeans. Examining the available evidence allows understanding of the rationale behind these decisions and thus a basis on which to create individualised treatment plans that take into account CVD risk, rate of projected eGFR decline, co-morbidities and other patient characteristics. BP goals are likely to change as an individual ages or develops more advanced disease. The management of BP in CKD should therefore be considered a dynamic process.

## Achieving BP Targets

Achieving BP targets is challenging. SPRINT demonstrated that, despite intensive input including monthly medication reviews, > 50% of those in the intensive treatment group failed to achieve the target systolic BP [50]. Results in those with CKD suggest that it may be even more challenging to achieve BP goals than in the general hypertension population [55]. Despite treatment with non-pharmacological interventions and multiple antihypertensive agents, the majority of CKD patients fail to reach target BP [56].

## Non-Pharmacological Treatment

There are a number of non-pharmacological treatments for hypertension in the context of CKD. A study by Slagman et al. [57] found that, in those already established on RAAS blockade, reducing dietary sodium intake to a target of < 50 mmol/day (~ 3 g/day of salt) decreased systolic BP by a further ~ 10 mmHg. A restriction to a target < 100 mmol/day (~ 6 g/day of salt) has also demonstrated a reduction in proteinuria by ~ 25%, an effect that is unlikely to be explained by BP reduction alone [58]. There are some in whom the effects of dietary sodium have little impact on BP. However, as GFR declines, the sensitivity of BP to dietary sodium load increases [17]. In practice, dietary sodium restriction can be difficult to achieve. Urinary sodium measurements by Slagman et al. [57] demonstrated that despite regular counselling sessions, the mean dietary sodium intake in the group targeting < 50 mmol/day was 106 mmol/day. In those without dietary restriction the intake was 186 mmol/day. Acknowledging personal dietary preferences and setting realistic goals (< 100 mmol/day of sodium) under the supervision of a dietitian may improve the likelihood of achieving meaningful and sustained dietary changes [59].

Weight loss is effective in reducing BP and proteinuria and may slow CKD progression [60]. In overweight patients (body mass index [BMI] > 27 kg/m^2^) with CKD and proteinuria (> 1 g/24 h), a mean weight loss of ~ 4% can reduce proteinuria by ~ 30% [61]. The benefits of a multidisciplinary approach have also been demonstrated in CKD. A systematic review by Santschi and colleagues [62] has shown improved attainment of BP goals in hypertensive patients following the introduction of community pharmacists. In the MASTERPLAN (Multifactorial Approach and Superior Treatment Efficacy in Renal Patients with the Aid of Nurse Practitioners) study conducted in the Netherlands, specialised nursing care clearly improved the management of CVD risk factors, including BP, at 1 and 2 years in patients with stage 3–4 CKD [63].

## Pharmacological Treatment

Despite the benefits of non-pharmacological interventions in CKD, antihypertensive medications are usually also required [56]. As well as direct BP-lowering effects, certain pharmacological therapies provide additional renoprotective and/or cardioprotective action, which may be independent of their BP-lowering effects [47]. The choice of drug should therefore consider the balance of risk reduction required by the individual. Combination drug therapy is frequently needed to achieve BP targets [64], and thus the risks of polypharmacy need also be considered.

### Renin–Angiotensin–Aldosterone System Blockade

ACE inhibitors and angiotensin II receptor antagonists (blockers) (ARBs) have both cardioprotective and renoprotective properties and are therefore of particular value in patients with CKD [42]. RAAS blockade can reduce systolic BP by ~ 20 mmHg in patients with hypertension and CKD [55]. This is similar to the BP reduction offered by CCBs and diuretics. Importantly, however, these agents offer a BP-independent reduction in proteinuria in both diabetic and non-diabetic CKD and are therefore generally accepted as first-line management of hypertension in patients with proteinuric CKD [39, 64–66].

In those with non-proteinuric CKD the superior renoprotective effect of RAAS blockade has recently been questioned. A systematic review carried out by Casas and colleagues [67] demonstrated that the improved renal outcomes associated with RAAS blockade are most likely due to a BP-lowering effect only, and could therefore be mirrored by other antihypertensives if the same reduction in BP was achieved. Consequently, although ACE inhibitors may be used as first-line agents in those with hypertension and non-proteinuric CKD, CCBs and thiazide or thiazide-like diuretics should also be considered as alternative first-line choices in this population [46].

In up to 50% of patients chronic ACE inhibition leads to angiotensin II reactivation with subsequent blunting of the efficacy of RAAS blockade. It has therefore been hypothesised that the addition of an ARB to those already established on ACE inhibition might improve cardiovascular and renal outcomes. ONTARGET (Ongoing Telmisartan Alone and in Combination with Ramipril Global Endpoint Trial) aimed to answer this question in patients at high risk of CVD [68]. 25,620 patients were randomised to either ACE inhibitor, ARB or combination therapy with both agents and followed up for a median period of 56 months. Combination therapy was associated with an increased incidence of adverse effects with no significant reduction in the primary outcome of death from CVD, MI, stroke or heart failure. The VA NEPHRON-D (Veterans Affairs Nephropathy in Diabetes) study examined renal outcomes in a cohort of diabetic patients with CKD [69]. Again, combination therapy with an ACE inhibitor and ARB led to an increase in adverse events without a significant reduction in the primary endpoint of progression of CKD, ESRD or death. Combination therapy with both an ACE inhibitor and ARB is therefore no longer advised in those with CKD. Notably, these studies included subjects at high risk of renal vascular disease, in whom GFR is to some extent dependent on a functional RAAS. In patients with conditions such as immunoglobulin A (IgA) nephropathy, the leading primary glomerular cause of ESRD in the USA, dual angiotensin blockade reduces proteinuria to a greater extent than monotherapy. These patients are younger and at lower cardiovascular risk than those in VA NEPHRON-D and ONTARGET. Whether this translates to a greater cardiovascular and renal benefit is currently unclear.

Potential problems associated with RAAS blockade include hyperkalaemia and the development of AKI. A rise in serum creatinine is often seen after initiation of RAAS blockade due to a reduction in intraglomerular pressure [70]. The RENAAL (Reduction in End Points in Noninsulin-Dependent Diabetes Mellitus with the Angiotensin II Antagonist Losartan) study randomised 1513 patients with diabetic nephropathy (defined as proteinuria > 0.5 g/day or serum creatinine 115–265 μmol/L) to losartan or placebo. The losartan group demonstrated a greater fall in eGFR during the first 3 months of treatment (– 2.3 vs – 1.6 mL/min/1.73 m^2^) [39, 71]. The significance of this transient fall in eGFR is unclear. Observational data from the UK primary care setting have demonstrated adverse cardiorenal outcomes in those with an initial eGFR decline following initiation of an ACE inhibitor or ARB [72]. In contrast, after a mean follow-up period of 3.4 years, RENAAL patients randomised to losartan had a slowed rate of eGFR decline compared with placebo regardless of initial rate of decline. Current guidelines suggest that a rise in serum creatinine of up to 30% with subsequent stabilisation should be accepted following initiation of RAAS blockade as this is likely to confer longer-term renoprotection [43].

Further uncertainty exists regarding the use of RAAS blockade in those with advanced CKD (eGFR < 30 mL/min/1.73 m^2^) as this population has been largely excluded from major randomised trials. An observational study by Ahmed et al. [73] demonstrated a significant increase in eGFR when RAAS blockade was withdrawn in a cohort of patients with advanced CKD in the UK, which in some cases prolonged time to starting renal replacement therapy. As a consequence, a national, multicentre, randomised trial of ACE inhibitor/ARB withdrawal (STOP-ACEi) in advanced CKD has begun [74]. Until results from this trial are available, the question of whether or not to initiate or continue RAAS blockade in those with advanced CKD remains uncertain.

### Diuretics

Volume overload, often subclinical, affects up to 50% of people with CKD and is an independent risk factor for CVD [75]. Diuretic therapy can reduce volume expansion and has been shown to improve left ventricular mass index and arterial stiffness in those with CKD [76, 77]. Thus, diuretics are frequently used as part of combination drug therapy in CKD and offer antihypertensive and cardioprotective effects [76].

In non-proteinuric CKD, monotherapy with a thiazide (such as bendroflumethiazide) or a thiazide-like diuretic (such as indapamide) may have a role and should be considered as a potential for first-line therapy [43]. Treatment with a diuretic may also reverse the loss of physiological nocturnal dip in BP described in CKD [78]. Loop diuretics (such as furosemide) are valuable, although higher doses are often required in those with a lower eGFR as the tubular mechanism of action of these drugs relies first on glomerular filtration. The combination of a loop and thiazide diuretic is particularly powerful, and care should be taken to avoid fluid depletion. Diuretics should generally be avoided in patients with polycystic kidney disease due to accelerated cyst growth and loss of excretory function associated with their use [79].

Mineralocorticoid receptor antagonists (blockers) (such as spironolactone) effectively reduce BP in CKD but run the risk of exacerbating hyperkalaemia [80]. These agents have been demonstrated to improve systolic and diastolic function in early CKD and therefore may be of particular value in patients with concomitant left ventricular dysfunction [81]. It is unclear whether this effect is due to BP lowering alone. In order to answer this question, a randomised study comparing spironolactone with the thiazide-like diuretic chlorthalidone in patients with CKD stage 3 has been completed (SPIRO-CKD [Spironolactone in Chronic Kidney Disease]) and results are awaited [82]. In hypertensive patients without CKD, spironolactone is more effective than bisoprolol or doxazosin at reducing BP when used as a fourth-line add-on therapy [83].

### Calcium Channel Antagonists (Blockers)

Both dihydropyridine and non-dihydropyridine CCBs are useful in the management of hypertension in CKD. Dihydropyridine CCBs (such as amlodipine) can be used as first-line therapy in non-proteinuric CKD, either alone or in combination. In proteinuric CKD their effect is inferior to RAAS blockade [41]. However, the addition of a dihydropyridine CCB to proteinuric patients with established RAAS blockade improves BP control without worsening proteinuria [39]. This has been reflected in the recently updated ESC/ESH guidelines which advocate combination therapy with an ACE inhibitor and CCB as first-line therapy in proteinuric patients [46]. Non-dihydropyridine CCBs (such as verapamil) have a superior effect on proteinuria reduction and are as effective as dihydropyridine CCBs in terms of BP control [84].

The ACCOMPLISH (Avoiding Cardiovascular Events through Combination Therapy in Patients Living with Systolic Hypertension) trial evaluated combination therapy with amlodipine and an ACE inhibitor versus hydrochlorothiazide and an ACE inhibitor in reducing CVD mortality in those with hypertension and at high risk of CVD (defined as the presence of diabetes, left ventricular hypertrophy, peripheral arterial disease, CKD or history of CVD) [85]. This multicentre, double-blind, randomised trial also included the prespecified endpoint of progression of CKD, defined as a doubling of baseline serum creatinine or reaching ESRD. The trial was terminated early due to superior efficacy of amlodipine and ACE inhibitor on CVD mortality. Notably, there was also a significantly lower risk of CKD progression in the amlodipine group that was independent of attained BP values. This suggests that the addition of amlodipine to ACE inhibitor therapy does exert an additional renoprotective effect over the addition of a thiazide diuretic in this at-risk group. Although generally well-tolerated, CCBs have the potential to worsen peripheral oedema, something that can be particularly troublesome for those with CKD [85].

### β-Blockers

β-Blockers (β-adrenoceptor antagonists) effectively reduce BP in CKD due to their effect on the dysregulated sympathetic nervous system [15]. The cardioprotective benefits of these drugs are well-established [86, 87] and therefore they are particularly advantageous in those with CKD. In animal studies, β-blockers also display renoprotective effects, including a reduction in the development of interstitial fibrosis following renal injury [88, 89]. Prospective and observational studies have demonstrated survival benefit from β-blocker therapy compared with placebo in patients with CKD [90, 91]. Despite this, β-blockers are used less frequently in those with than in those without CKD [92]. Underuse in patients with CKD may be partially explained by concerns regarding glycaemic control, reduced renal excretion and systemic accumulation [93, 94]. Although these are potential risks with certain classes of β-blockers, these drugs can be safely used in all degrees of renal impairment. Dosing adjustments may be required, and hepatically excreted β-blockers and those with additional vasodilatory properties (such as carvedilol) are likely to be of particular value [95]. Direct comparisons with ACE inhibitors have shown β-blockers to offer inferior renoprotection [96, 97]. The AASK study did, however, demonstrate lower rates of ESRD and death in CKD patients treated with metoprolol versus amlodipine [47]. β-Blockers should therefore be considered as useful additions in those with established RAAS blockade, particularly when overt CVD coexists.

### α-Blockers

Peripherally acting α-blockers (α-adrenoceptor antagonists; such as doxazosin) are commonly used as part of combination therapy for the management of hypertension in CKD. This may be due to a pharmacokinetic profile that is undisturbed by declining eGFR in addition to favourable effects on glycaemic control [98]. Several studies have demonstrated their efficacy as add-on therapy in the management of hypertension in CKD [99, 100]. α-Blockers should not, however, be considered for first-line therapy, as they are less effective than other agents for reducing the incidence of CVD [101].

### Chronotherapy

As the diurnal variation of BP can be influenced by timing of antihypertensive medications, it has been hypothesised that evening dosing could reverse the non-dipping nocturnal BP seen in CKD. A study by Hermida and colleagues [102] explored the effects of nocturnal antihypertensive dosing in 661 patients with CKD by assigning participants to take all antihypertensive medication upon wakening or to take one or more antihypertensive at night. After a median follow-up period of 5.4 years the nocturnal dosing group had significantly better BP control and lower incidence of CVD death, MI and stroke than those who took all medications in the morning. Chronotherapy would therefore seem to be one of the more straightforward methods of achieving improved outcomes for those with hypertension and CKD.

### Adherence

Despite the risks of CKD progressing to ESRD and patients requiring dialysis and/or transplantation, adherence to therapy is no better in those with CKD than in those without [103]. Adherence improves as CKD advances but deteriorates again as patients start dialysis [104]. Studies examining the reasons for non-compliance in those with CKD highlight the importance of communication and perceived benefit of the therapies in question [105]. Pill burden, drug interactions and adverse effects are also important. Antihypertensive regimens should therefore be simplified wherever possible, with consideration given to the quantity, timing and formulation of interventions. Continuity of care may also have an impact and, where possible, attempts should be made to allow patients to see the same clinician at each visit, something that has been demonstrated to improve outcomes [106].

## Managing Hypertension in the Context of Haemodialysis

The relationship between BP and CVD outcomes in patients undergoing haemodialysis is complex. In this cohort, a lower BP does not necessarily translate into improved survival, as it does in the general population and those with pre-dialysis CKD [107, 108]. Evidence to guide BP targets in this group is limited. In a study by Li and colleagues [108], 125,928 haemodialysis patients were grouped into different pre-dialysis systolic BP categories and observed over 3 years. The highest mortality was seen in those patients with a pre-dialysis systolic BP of < 120 mmHg. A higher pre-dialysis BP was protective, with the lowest mortality seen in those with a pre-dialysis systolic BP of 160–180 mmHg [108]. This ‘inverse epidemiology’ has been demonstrated previously, although remains poorly understood [109]. Changes in BP during haemodialysis may also predict adverse clinical outcomes. Retrospective data have demonstrated that intradialytic hypertension is associated with an increased 30-day mortality and rate of hospitalisation [110].

The optimal way to measure BP in patients undergoing haemodialysis is debatable. Large shifts in fluid and electrolytes mean pre- and post-dialytic BPs may differ significantly. Mayer et al. [111] utilised 24-h ABPM applied immediately before a dialysis session to examine the relationship between systolic BP and mortality in a haemodialysis cohort. During the 3-year follow-up period a U-shaped relationship between 24-h systolic ABMP and all-cause mortality was evident, demonstrating an increased risk of death for those with either very low or very high BP. Subgroup analysis demonstrated a linear relationship in those with and without CVD. In patients with CVD, a lower BP was associated with a higher risk of death. The opposite was true for those without CVD, explaining the overall U-shaped curve. This demonstrates the need for personalised BP treatment plans that incorporate co-morbidities in patients undergoing haemodialysis. CVD risk calculators have long been available to predict the risk of CVD events for individual patients. Not only do novel risk calculators such as QRISK^®^3 now include those with CKD, but several risk calculators designed for use in the haemodialysis population have now been developed and validated [112, 113].

Hypertension in patients undergoing haemodialysis may be largely driven by sodium and water overload. However, hypertension often persists despite aggressive ultrafiltration [114]. All classes of antihypertensive may be used, although data governing this are limited. Use of β-blockers is particularly attractive as they mitigate some of the arrhythmogenic effects of dialysis and reduce arterial stiffness and left ventricular hypertrophy, both of which are accelerated in ESRD [95, 115]. Choice of β-blocker remains contentious, in part due to variable degrees of drug clearance during haemodialysis [95].

## Managing Hypertension Following Kidney Transplantation

**Fig. 3:**
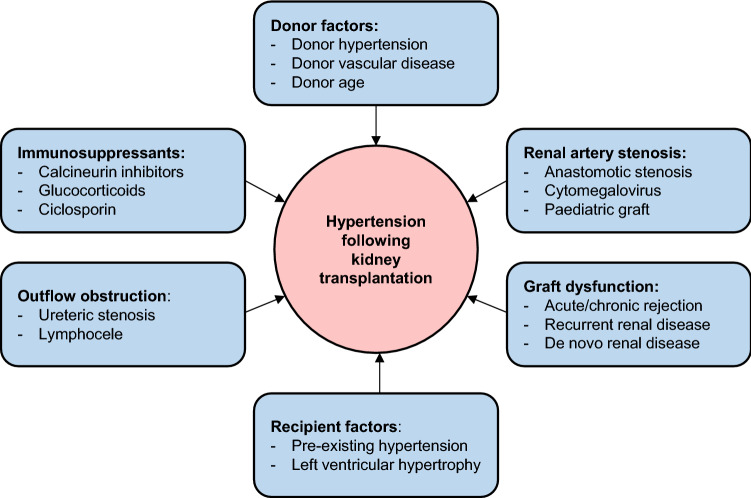
Factors contributing to the development of hypertension following kidney transplantation

As in haemodialysis, there are currently no randomised clinical trials exploring how best to manage hypertension following kidney transplantation. Hypertension is common post-transplant, with multiple factors contributing to its development (Fig. 3). More than 90% of recipients of a kidney transplant receiving a calcineurin inhibitor (CNI)-based immunosuppression regimen will be hypertensive post-transplant [116]. BP is also more likely to be uncontrolled, with ~ 50% failing to achieve a systolic BP < 140 mmHg at 1 year [117]. Higher BP is associated with poorer graft outcomes and greater CVD risk, which is the leading cause of death following kidney transplant [118–120]. A retrospective study of 1666 kidney transplant recipients demonstrated a ~ 5% increased risk of graft failure and death with every 10 mmHg rise in systolic BP [117]. Broader cardioprotection including lipid-lowering and antiplatelet therapy is likely to be beneficial in most patients [121].

Choice of antihypertensive agent will depend on several factors, including time from transplantation, concomitant prescriptions including immunosuppression, and co-morbidities. Historically, RAAS blockade has been avoided in the early post-transplant period due to the potential for a transient rise in serum creatinine at this precarious time. Despite this, a systematic review by Jennings and Taber [122] has suggested that early initiation of RAAS blockade (within 12 weeks post-transplant) improves left ventricular hypertrophy and proteinuria at 1 year without causing a fall in eGFR. Whether initiated early or not, these agents are considered to be important following renal transplantation due to the increased risk of CVD in this group [123]. However, there remains uncertainty regarding post-transplant renoprotection. A study by Knoll et al. [124] aimed to address this uncertainty by examining the effect of ramipril versus placebo in 212 transplant recipients with proteinuria at least 3 months post-transplant. After a follow-up period of 48 months, ramipril did not lead to a significant reduction in doubling of serum creatinine, ESRD or death. This uncertainty is reflected in low rates of ACE inhibitor use in this group, with as few as 30% of patients with CVD and/or diabetes receiving RAAS blockade 6 months post-transplant [121].

CNIs such as tacrolimus, which form the mainstay of post-transplant immunosuppression, cause afferent arteriolar vasoconstriction. There is some evidence to suggest that dihydropyridine CCBs confer particular benefit in those treated with CNIs due to their ability to cause afferent arteriolar vasodilatation in this context [125]. Non-dihydropyridine CCBs interfere with CNI metabolism, necessitating closer monitoring of drug levels [126].

## Future Directions

As populations live longer and the prevalence of diabetes increases, the global burden of CKD is likely to grow [127]. Managing elderly patients with hypertension and CKD is therefore an area of significant importance. Results from studies of elderly hypertensives without CKD demonstrate significant reductions in heart failure, stroke and death from CVD when BP is controlled [128, 129]; however, evidence in those with CKD, a group at higher CVD risk, is lacking. Future work should aim to enhance our understanding of optimum BP targets in elderly patients with CKD for both cardio- and renoprotection. The STOP-ACEi trial may provide useful information regarding the specific merits of RAAS blockade in this group [74].

Improved survival of patients with several chronic diseases has also led to an increased incidence of CKD in these groups. For example, the life expectancy of a 20-year-old patient starting antiretroviral therapy (ART) for HIV in the UK is now almost 70 years [130]. HIV is associated with an increased prevalence of both hypertension and CKD [131]. In those treated with ART aged > 50 years, more than 50% will be hypertensive, with an approximately four times higher prevalence of microalbuminuria than the general population [132]. Although the advent of ART has reduced the number of patients progressing to ESRD, there is still an excess mortality in those with ESRD secondary to HIV-associated nephropathy [133]. Efforts to identify and control hypertension and renal dysfunction in this patient group are therefore critically important.

There remains an unmet need for therapeutic options capable of slowing progression of CKD and attenuating the associated CVD risk. Interest in sodium–glucose cotransporter 2 inhibitors (SGLT2i), such as empagliflozin, has intensified following the EMPA-REG OUTCOME (BI 10773 [Empagliflozin] Cardiovascular Outcome Event Trial in Type 2 Diabetes Mellitus Patients) trial, which demonstrated significant slowing of CKD progression and a reduction in the composite outcome of death from CVD, non-fatal MI or non-fatal stroke in patients with type 2 diabetes and high CVD risk randomised to empagliflozin versus placebo [134, 135]. Exploratory studies have also suggested benefit from endothelin receptor antagonists in reducing both BP and proteinuria in those with CKD [136]. Although larger randomised studies have shown limited antihypertensive efficacy [137], DUET (Efficacy and Safety of Sparsentan [RE-021], a Dual Endothelin Receptor and Angiotensin Receptor Blocker, in Patients with Focal Segmental Glomerulosclerosis [FSGS]: A Randomized, Double-blind, Active-Control, Dose-Escalation Study) did demonstrate a reduction in proteinuria in patients with FSGS randomised to dual endothelin-A and angiotensin II receptor antagonists compared with ARB alone [138]. Phase III trials involving patients with both FSGS and IgA nephropathy are now underway. Direct renin inhibitors have shown promise in early studies but adequately powered, high-quality trials are required [139]. The prevalence of treatment-resistant hypertension in CKD is a growing concern and the inclusion of this patient group in large-scale randomised outcome trials may help to guide treatment [140, 141]. Interest in renal denervation for resistant hypertension has also been renewed following results from a proof-of-concept randomised trial [142]; however, significant doubt still exists as to whether this treatment is effective and safe in CKD.

Finally, there is a growing body of evidence to suggest that health literacy has a direct influence on outcomes in CKD [143]. As the global burden of this condition increases, targeting improvements in health literacy at both a local and national level may improve outcomes in this patient group. Practically, encouraging shared decision-making and patient involvement wherever possible might be a useful first step.

## Conclusion

Lowering BP in CKD slows disease progression and reduces incident CVD. An understanding of the pathophysiological mechanisms leading to the development of hypertension in this patient group is useful in order to effectively target both endpoints. Existing guidelines do not offer a consensus on optimal BP targets but are created based on evidence favouring interventions for either renoprotection, cardioprotection or both. Similarly, pharmacological therapies designed to achieve these targets offer different degrees of risk reduction based on patient characteristics. One size does not fit all, therefore, and an appreciation of what is achievable with BP reduction in CKD is key to making informed, individualised decisions.
