# Correction to: Management of Hypertension in Chronic Kidney Disease

**DOI:** 10.1007/s40265-020-01388-8

**Published:** 2020-08-25

**Authors:** Dan Pugh, Peter J. Gallacher, Neeraj Dhaun

**Affiliations:** 1 University/BHF Centre for Cardiovascular Science, The Queen’s Medical Research Institute, University of Edinburgh, 47 Little France Crescent, Edinburgh, EH16 4TJ Scotland UK; 2 Department of Renal Medicine, Royal Infirmary of Edinburgh, Edinburgh, UK

## Correction to: Drugs (2019) 79:365–379 10.1007/s40265-019-1064-1

This article was originally published under a CC By-NC 4.0 license, but has now been made available under a Creative Commons Attribution 4.0 International License (https://creativecommons.org/licenses/by/4.0/), which permits use, sharing, adaptation, distribution and reproduction in any medium or format, as long as you give appropriate credit to the original author(s) and the source, provide a link to the Creative Commons licence, and indicate if changes were made.

The original article has been corrected.

